# Immobilization of Air-Stable Copper Nanoparticles on Graphene Oxide Flexible Hybrid Films for Smart Clothes

**DOI:** 10.3390/polym14020237

**Published:** 2022-01-07

**Authors:** Peng-Yang Huang, Chen-Yang Huang, Jia-Wun Li, Sheng-Yen Shen, Chih-Chia Cheng, Chih-Wei Chiu, Ru-Jong Jeng, Jiang-Jen Lin

**Affiliations:** 1 Institute of Polymer Science and Engineering, National Taiwan University, Taipei 10617, Taiwan; d02549006@ntu.edu.tw (P.-Y.H.); reggieshen7@gmail.com (S.-Y.S.); 2 Department of Materials Science and Engineering, National Taiwan University of Science and Technology, Taipei 10607, Taiwan; d10504015@gapps.ntust.edu.tw (C.-Y.H.); a12352335@gmail.com (J.-W.L.); 3 Graduate Institute of Applied Science and Technology, National Taiwan University of Science and Technology, Taipei 10607, Taiwan; cccheng@mail.ntust.edu.tw; 4 Department of Materials Science and Engineering, National Chung Hsing University, Taichung 40227, Taiwan

**Keywords:** copper nanoparticles, graphene oxide, polymeric dispersant, nanohybrids, electrical conductivity, electrocardiogram, smart clothes

## Abstract

Through the use of organic/inorganic hybrid dispersants—which are composed of polymeric dispersant and two-dimension nanomaterial graphene oxide (GO)—copper nanoparticles (CuNPs) were found to exhibit nano stability, air-stable characteristics, as well as long-term conductive stability. The polymeric dispersant consists of branched poly(oxyethylene)-segmented esters of trimellitic anhydride adduct (polyethylene glycol−trimethylolpropane−trimellitic anhydride, designated as PTT). PTT acts as a stabilizer for CuNPs, which are synthesized via in situ polymerization and redox reaction of the precursor Cu(CH_3_COO)_2_ within an aqueous system, and use graphene oxide to avoid the reduction reaction of CuNPs. The results show that after 30 days of storage the CuNPs/PTT/GO composite film maintains a highly conductive network (9.06 × 10^−1^ Ω/sq). These results indicate that organic/inorganic PTT/GO hybrid dispersants can effectively maintain the conductivity stability of CuNPs and address the problem of CuNP oxidation. Finally, the new CuNPs/PTT/GO composite film was applied to the electrocardiogram (ECG) smart clothes. This way, a stable and antioxidant-sensing electrode can be produced, which is expected to serve as a long-term ECG monitoring device.

## 1. Introduction

Through the use of wearable smart clothes, heart rhythm, heartbeat, blood pressure, pulse, and other health factors can be monitored and displayed on a computer screen [1,2]. Cardiovascular disease accounts for nearly half of all deaths worldwide [3]. An electrocardiogram (ECG) is often employed to observe and monitor a patient’s heart health [4] and its characteristic peak signals can be used to identify symptoms of arrhythmia. Commercially available Ag/AgCl electrodes can generally serve as detectors for ECG physiological signals. However, they cannot be reused and tend to cause skin allergies and other problems [5]. Studies have shown that metallic silver [6], stainless steel wire [7], conductive polymer [8], carbon-based nanomaterial [9], or other composite materials can be used as alternative materials for dry electrodes [10,11]. These materials can be used in place of wet Ag/AgCl electrodes for the detection of ECG physiological signals.

CuNPs have a high surface activity, making them susceptible to oxidation in air. However, compared with precious metal materials such as gold and silver, CuNPs have the advantage of being inexpensive and simple to prepare. CuNPs have attracted considerable research attention for various applications, including in electrical and thermal conductivity materials [12], semiconductor components [13], solar cells [14,15], capacitors [16,17], buildings [18], and electronic devices [19,20]. The most common methods for preparing CuNPs include the gas phase method [21,22], the solid phase method [23,24], and the liquid phase method [25,26]. Equipment employed in the gas phase method is expensive and difficult to operate; particles obtained by the solid phase method have rough surfaces and uneven particle size distributions. In comparison, the liquid phase method has low equipment requirements, allows easy control of particle size, and a high production efficiency, which have led to its wide applications in research [27,28]. For CuNPs, the smaller particle size leads to a higher surface activity and a greater probability of agglomeration and air oxidation [29]. Thus, it is essential to control the synthesis conditions and particle uniformity during the preparation of CuNPs. As part of the synthesis and manufacturing process, inert gas is often used to protect the CuNPs from surface oxidation and to control their particle size, which significantly increases manufacturing costs. Several studies have proposed the use of surfactant polymers to cover the CuNPs surface to solve the problems of oxidation and agglomeration [30]. Microstructurally, the hydrophilic and hydrophobic groups of surfactants can lead to the formation of spherical nano-micelles, typically with nanoscale diameters. These nano-micelles can be coated on the surface of the nanometal to prevent agglomeration [31]. Generally, a dense molecular structure of surfactants is required to prevent oxygen from contacting nanometals [32,33,34]. Chen et al. prepared CuNPs/graphite composites and explored the effects of three surfactants on the size uniformity and electrochemical behaviors of CuNPs [35]. Hosseini et al. investigated the effects of surfactants on the hydrogen evolution reaction (HER) of CuNPs. Three surfactants, namely Triton X-100, cetyltrimethylammonium bromide (CTAB), and sodium dodecyl sulfate (SDS), were used as protective agents for CuNPs, and CuNPs-nanocarbon hybrid materials were successfully prepared. The hybrid material prepared with SDS as the protective agent exhibited the best electrochemical activity [36]. Amorphous carbon and graphene-based materials have also been used in many studies to reduce the agglomeration of CuNPs [37,38]. Yang et al. examined the use of CuNP/graphene oxide (GO)/single-walled carbon nanotube (SWCNT) hybrid material as an electrochemical-sensing platform for nonenzymatic glucose detection. With the addition of GO and SWCNT, conductivity was increased and dispersibility was improved, CuNPs became more active and excellent glucose detection capabilities were provided [39]. Graphene has also been more often used in wearable devices in recent years and has become one of the easiest materials to use in wearable sensing technology. For example, the use of CVD technology to grow graphene on PDMS substrate to make flexible electrodes for ECG devices, sensors for interactive human-machine interface (iHMI) systems, and motion detection or environmental monitoring devices have been prepared, which shows that performance of materials in wearable devices is constantly being improved in future use [40]. In the past few years, our research group has also successfully synthesized highly flexible and conductive nanohybrid electrode films. Electrodes using the composition of nanosilver/dispersant/GO have demonstrated high conductivity. However, silver is expensive and the characteristics of air oxidation have not been addressed in detail [6,41,42,43], while copper nanoparticles are hindered by safety concerns. Studies have shown that in 2013, Cohen et al. [44] found that the toxicity of CuNPs firstly penetrate directly into cells from CuNPs, and secondly, Cu^2+^ ions diffuse into the cytoplasm. In 2019, Zanoni et al. [45] conducted a study on the penetration of CuO into the skin. They showed that when CuNPs make contact with intact skin epidermis, there were no safety concerns, while damaged skin surfaces showed Cu^2+^ or CuNPs penetration concerns; revealing CuNPs will not pose any safety concerns for intact human skin. As mentioned above, it is known that CuNPs may cause inflammation on the surface of damaged skin or in contact with acidic sweat. Therefore, the low melting point characteristics of nano-scale particles can be used for sintering to prepare the interconnected network structure [6], thereby increasing safety and conductivity. For example, Kwon et al. used Cu to manufacture wearable flexible hybrid electronic products, while attached to the skin to detect physiological signals [46].

Numerous studies have utilized high-conductivity metals as electrodes for ECG smart clothes; however, factors such as cost and susceptibility to air oxidation must be considered when choosing materials. In this study, low cost CuNPs were chosen as the electrode material for ECG smart clothes. A hybrid material was developed by blending the self-designed and synthesized polymeric dispersant with graphene. This hybrid material can be used to stabilize CuNPs, control the nanoparticle size, and provide high air oxidation stability for CuNPs. The hybrid electrode film exhibits a resistance comparable to that of typical copper foil at room temperature. In addition, it can effectively inhibit the surface oxidation of CuNPs, thereby enabling the monitoring and recording of ECG physiological signals from the human body over an extended period of time.

## 2. Experimental

### 2.1. Materials

Graphene oxide (GO) was purchased from Enerage lnc., Yilan County, Taiwan. NaBH_4_ and nitric acid (HNO_3_, 65%) were purchased from Merck KGaA Inc., Taipei, Taiwan. Polyethylene glycol, (PEG Mw = 1000 and 20,000) was purchased from Alfa Aesar, MA, USA. The compound 1,1,1-Trimethylolpropane (TMP) was purchased from Acros, MA, USA. Trimellitic anhydride (TMA) was purchased from Tokyo Chemical Industry Co. Ltd., Tokyo, Japan. Cu(CH_3_COO)_2_·H_2_O was purchased from SHOWA Chemical co. Ltd., Tokyo, Japan. The reagents listed above are all of high purity and can be used without further purification.

### 2.2. Synthesis of Branched Polyester Polymeric Dispersant

**Figure 1:**
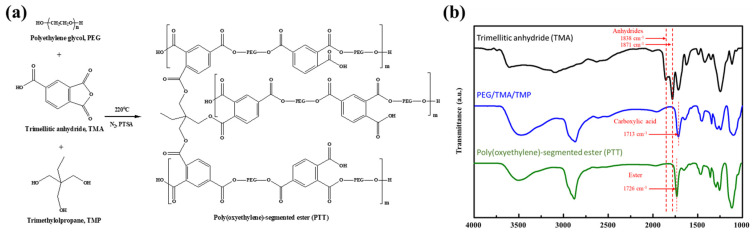
(**a**) Synthetic scheme for the branched polymer dispersant—Polyethylene glycol-trimethylolpropane-trimellitic anhydride (PTT). (**b**) FTIR spectrum analysis of the PTT polymeric dispersant.

Based on our previous study [47], the branched polyester polymeric dispersant is a block segmented polyether ester synthesized with PEG1000 (diol), TMP (triol), and TMA (triacid) at a molar ratio of 3:1:3. The result consists of branched poly(oxyethylene)-segmented esters of trimellitic anhydride adduct that form polyethylene glycol-trimethylolpropane-trimellitic anhydride, referred to here as PTT. The synthesis steps are illustrated in Figure 1a. First, PEG-1000 (30 g, 60 mmol) and TMP (1.34 g, 20 mmol) were added into a 250 mL three-necked round-bottomed flask equipped with a mechanical stirrer, nitrogen lines, and a thermometer. The mixture was then heated to 80 °C to ensure thorough mixing. Next, TMA (5.77 g, 60 mmol) was added to the reactants and gradually heated to 160 °C and maintained for 3 h to remove water generated from esterification through the trap. Finally, a slightly yellowish crystalline solid product was produced at room temperature. Fourier-transform infrared spectroscopy (FTIR) was used to analyze the mixture. The FTIR spectra of PTT are presented in Figure 1b. Acid anhydride rings were observed at 1781 and 1838 cm^−1^ in the TMA spectrum. After the addition of PEG and TMP, the anhydride ring disappeared and the characteristic absorption for ester formation at an elevated temperature was observed at 1726 cm^−1^. During the reaction at 160 °C, the formation of the largest ester functional group was observed at 1713 cm^−1^ corresponding to the carboxylic acid.

### 2.3. Preparation of the CuNPs/PTT/GO Nanohybrids

First, PTT was added to DMF and H_2_O (*w*/*w* = 1:1) to form a hydrolyzed PTT solution. Next, Cu(CH_3_COO)_2_·H_2_O was added to DMF and H_2_O (*w*/*w* = 1:1). The above PTT solution was added to the CuNPs solution at a weight ratio of 1:1 and then stirred magnetically until complete dissolution. The GO was gradually added to the prepared solution to form CuNPs/PTT/GO samples with weight ratios of 1:1:0, 20:20:1, 10:10:1, 5:5:1, and 1:1:1. The mixture was then agitated using an ultrasonic oscillator for 30 min and mixed continuously for 3 h using a magnetic stirrer at 80 °C. Following this, sodium borohydride (NaBH_4_) was added as a reducing agent. After 1 h in an ice bath, a change was observed in the color of the uniform CuNPs mixture from blue-green to wine-red. The reduction status was determined using UV-vis spectroscopy and *X*-ray, and the particle size was observed using a transmission electron microscope (TEM) and calculated by TEM image.

### 2.4. Preparation of the CuNPs/PTT/GO Film Electrode

The CuNPs/PTT/GO solution was centrifuged to obtain the bottom precipitate, which was applied to the filter membrane surface using an automatic coater to form a 50-μm-thick film. The film was then placed in an oven and slowly heated to 80, 150, 250, and 300 °C for melting. During this process, nitrogen was injected to prevent the oxidization of copper particles. A four-point probe was used to measure the conductivity of the CuNPs/PTT/GO conductive film during the heating process, and a high-resolution field emission scanning electron microscope (FE-SEM) was used to observe its surface. Finally, two equal-sized (6 cm × 3 cm) electrodes of CuNPs/PTT/GO were cut from the sintered filter membrane. Metal buttons were added to the CuNPs/PTT/GO electrode and connected to the ECG sensor to measure ECG signals.

### 2.5. Characterization and Instruments

The PTT dispersant was analyzed using a PerkinElmer Spectrum One FTIR spectrometer, which has a test range of 1000 to 4000 cm^−1^. The reduction reaction of CuNPs was observed using a JASCO V-630 UV-vis spectrophotometer (JASCO Corporation, Kyoto, Japan) with a test range of 400 to 800 cm^−1^, where the CuNPs/PTT/GO solution was diluted 1/100 times. The TEM (Zeiss EM 902A, Oberkochen, Germany) was used to observe the CuNPs particle size in the diluted CuNPs/PTT/GO solution. The average particle size of CuNPs was analyzed using the ImageJ software. The precipitate was obtained using the centrifuge (Z206A, Hermle Labortechnik GmbH, Wehingen, Germany), which was run at 6000 rpm for 1 hr. After being dried at 80 °C, the CuNPs/PTT/GO film was imaged using a high-resolution FESEM (JEOL-JSM 6500F, JEOL, Ltd., Tokyo, Japan), and the *X*-ray pattern was acquired by *X*-ray diffraction (XRD, Shimadzu SD)-D1) Cu Kα radiation (35 kV, 30 mA). A four-point sheet resistance meter (Four-point probe, MCP-T610, Mitsubishi Chemical Corporation, Kanagawa, Japan) was used to evaluate the conductivity of all conductive films. A lab-designed LED bulb circuit was used to conduct the illumination test for all CuNPs/PTT/GO films. The ECG Device (TD3, Yang Yin Co. Ltd., Taipei, Taiwan) employed the bipolar method to record the P-Q-R-S-T waveform of the ECG. The ECG records obtained using CuNPs/PTT and CuNPs/PTT/GO films over a course of 30 days were compared. The washing test uses AATCC test method 135 and the Maytag washing machine (MAT14PD) for 20 machine washing cycles, the test sample size 5 × 5 cm is square.

## 3. Results and Discussion

### 3.1. Dispersion Characteristic of CuNPs/PTT/GO Nanohybrids

**Figure 2:**
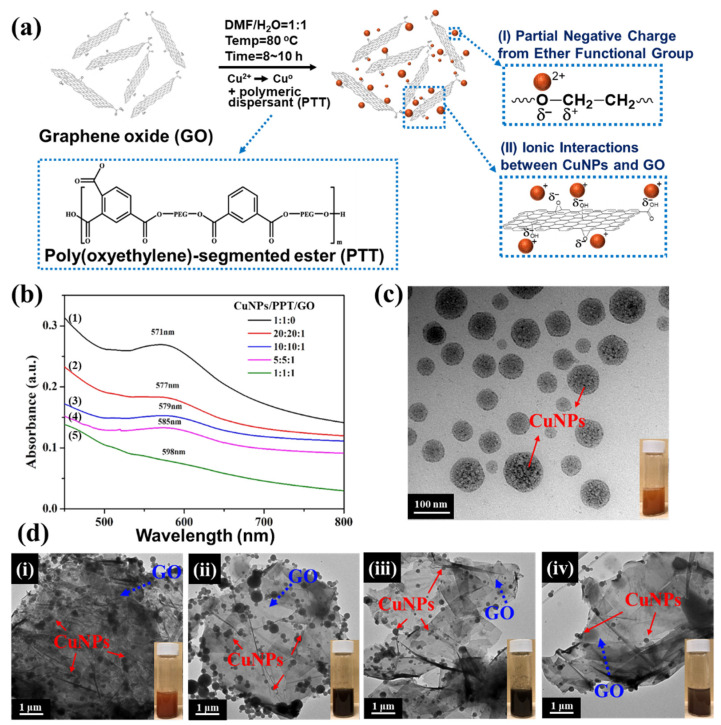
(**a**) Schematic illustration of the in situ reduction and dispersion mechanism of CuNPs on GO. (**b**) UV-vis spectrum of CuNPs/PTT/GO, CuNPs/PTT/GO ratio. (1) 1:1:0, (2) 20:20:1, (3) 10:10:1, (4) 5: 5:1 and (5) 1:1:1. (**c**) TEM and digital images of reduced CuNPs when mixed with PTT at a ratio of 1:1 and without GO. (**d**) TEM and digital images of reduced CuNPs/PTT/GO with a weight ratio of (i) 20:20:1, (ii) 10:10:1, (iii) 5: 5:1, and (iv) 1:1:1.

**Figure 3:**
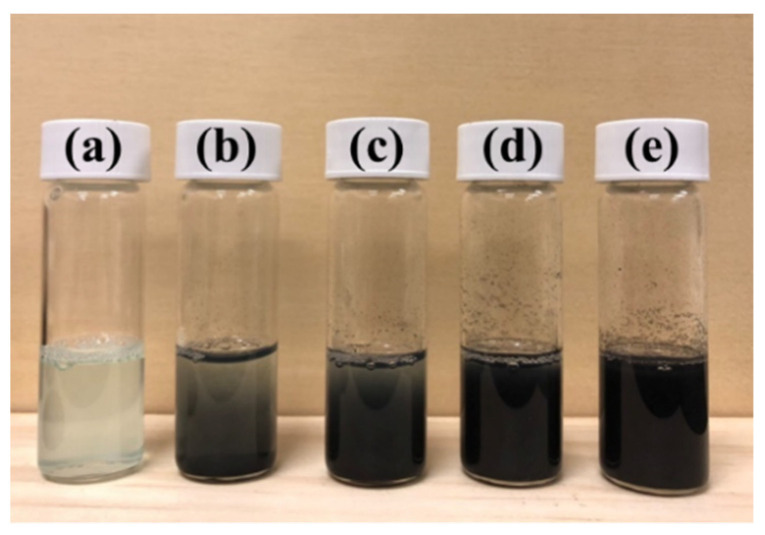
Digital photos of CuNPs/PTT/GO hybrid dispersions before reduction, the ratio of CuNPs/PTT/GO (**a**) 1:1:0, (**b**) 20:20:1, (**c**) 10:10:1, (**d**) 5:5:1, and (**e**) 1:1:1.

According to our previous research, the polymeric dispersant PTT can be synthesized using PEG1000, TMP, and TMA [47]. The design concept for the polymeric dispersant PTT involves utilizing the non-covalent bond between PEG and CuNPs on the main chain segment of the molecule to absorb the nanoparticles, and then perform an in situ reduction reaction [48]. During the synthesis reaction, Cu^2+^ can be converted to Cu^0^. Through noncovalent van der Waals force and ionic-charge interactions of the noncovalent bond, CuNPs can be stabilized by adsorption to the polymer. As a result of the non-covalent bonds between polymers, CuNPs and PTT exhibit excellent compatibility due to their mutual force. In addition, other forces—such as the interactions between the trimellitic anhydride group on TMA and the carboxyl, hydroxyl, and epoxy groups on GO—allow for CuNPs to adsorb onto the surface of GO. The complete reaction steps are presented in Figure 2a. Furthermore, GO can form an electrostatic attraction force with Cu^2+^ through the electronegativity of oxygen-containing functional groups on its surface. This force can adsorb copper ions on the surface of GO, thereby further reducing Cu^2+^ to achieve nanoscale stabilization of CuNPs. Further, the oxygen atoms within the branched polyester polymeric dispersant PTT can exert an interaction force with Cu^2+^ to allow further stabilization of the CuNPs/PTT/GO nano mixture solution. The UV-vis spectra of the samples following the reduction reaction are presented in Figure 2b. Upon reduction, the CuNPs exhibited a characteristic absorption peak at 570–590 nm of the UV-visible spectrum. Note that a peak will not appear at 570–590 nm if the reduction of CuNPs has not been completed. The increase in the peak intensity of curve (1) indicates that CuNPs have formed a particle state. In curves (2–5), the intensity of the absorption peaks decreased markedly as the concentration of GO increased. The results indicate that the surface of CuNPs was gradually covered by GO. At last, the absorption peak in curve (5) disappeared, indicating that PTT effectively adsorbed CuNPs onto the GO surface and formed CuNPs/PTT/GO. TEM was used to characterize the morphology and size of CuNPs/PTT/GO. Figure 2c shows the TEM and digital images of reduced CuNPs when mixed with PTT at a ratio of 1:1 and without GO. As can be seen in the digital image, the CuNPs/PTT solution appeared reddish-brown without GO. The TEM image shows that PTT caused CuNPs to be spherical, and the PTT coating on the surface of the CuNPs can be seen clearly. The effects of ratios of CuNPs/PTT/GO being (i) 20:20:1, (ii) 10:10:1, (iii) 5:5:1, and (iv) 1:1:1 on the stability of CuNPs and the corresponding digital images are presented in Figure 2d. As the proportion of GO increased, it was observed that the number of CuNPs on the surface of GO gradually decreased, and the size of the 2D GO flake was 3–5 μm. In the absence of GO, relatively large particles of CuNPs, with an average size of 35–100 nm, were produced. With the addition of GO, the average particle size of CuNPs was reduced to approximately 10–35 nm. CuNPs were also observed to be arranged between the GO layers, forming a CuNPs/PTT/GO material with a good 3D structure. The digital images of solutions showed that the GO-containing PTT appeared dark red after being reduced with NaBH_4_. It was noted that each sample displayed a slightly different color, which may be due to the particle size of CuNPs when reduced under different conditions. The digital images of the samples before reduction are presented in Figure 3. As can be seen, when Cu(CH_3_COO)_2_·H_2_O was dissolved in DMF and H_2_O (*w*/*w* = 1:1), the solution appeared light blue. In this observation, it is again demonstrated that Cu^2+^ was successfully reduced to Cu^0^ following the reduction by NaBH_4_.

### 3.2. Preparation and Surface Morphology of CuNPs/PTT/GO Composite Films

**Figure 4:**
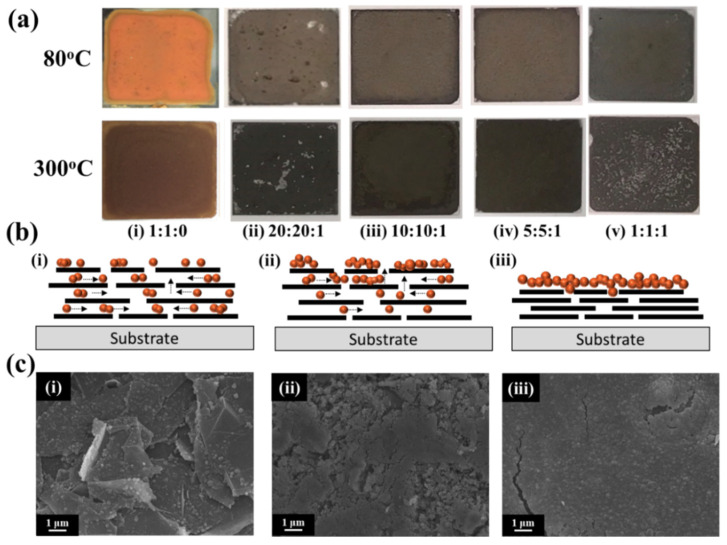
(**a**) Digital images of CuNPs/PTT/GO hybrid films (2 cm × 2 cm) at different ratios: (i) 1:1:0, (ii) 20:20:1, (iii) 10:10:1, (iv) 5:5:1, and (v) 1:1:1. (**b**) Schematic diagram and (**c**) SEM images of conductive film formed by high temperature sintering of CuNPs/PTT/GO. FE-SEM images of CuNPs/PTT/GO films under different temperatures: (i) 150 °C, (ii) 250 °C, and (iii) 300 °C.

To further confirm the surface resistance characteristics of CuNPs/PTT/GO, the solution was made into a conductive film. The CuNPs/PTT/GO dispersion solution was first centrifuged at room temperature; then, the CuNPs/PTT/GO was removed from the fluid and coated on the glass surface. Next, a staged heating program was performed at 80 °C, 160 °C, 250 °C, and 300 °C. The temperature was held for one hour at each stage to melt and sinter the sample, as shown in Figure 4. It can be seen from Figure 4a that CuNPs/PTT/GO exhibited a change in color after high-temperature sintering. Sample (1) changed from orange to wine red after sintering at a high temperature. Samples (2–5) showed little color change with increasing GO concentration, and all appeared graphite in color. Following high-temperature sintering, samples (1), (3), and (4) formed continuous thin films, with the 10:10:1 weight ratio producing the lowest resistance of 8.12 × 10^−2^ Ω/sq; samples (2) and (5), on the other hand, produced discontinuous holes, causing their surface resistance to increase to 3.45 × 10^0^ Ω/sq and 4.27 × 10^1^ Ω/sq, respectively. The schematic diagram of the conductive film made from CuNPs/PTT/GO is illustrated in Figure 4b. As nanometals have a low melting point, heat treatment was used to melt CuNPs, enabling them to float to the surface through the gaps in the GO. FE-SEM was then employed to further observe the changes in the surface of the CuNPs/PTT/GO = 10:10:1 film. It can be seen from Figure 4c-(i) that the copper particles migrated to the film surface as the temperature increased. Figure 4c-(ii) shows that the CuNPs aggregated onto the polymer chain to form an interconnected network structure, and the PTT disappeared at an elevated temperature. As shown in Figure 4c-(iii), when the temperature reached 300 °C, the remaining CuNPs/GO film formed a continuous film. The average particle size of CuNPs, UV-vis absorption peak intensity, and surface resistance after sintering are listed in Table 1. According to the results, when GO was not added to CuNPs/PTT, the resistance was 6.92 × 10^−2^ Ω/sq after heat treatment. Upon addition of GO and heat treatment, the CuNPs/PTT/GO with ratios of 10:10:1 and 5:5:1 exhibited a resistance similar to CuNPs/PTT. The lowest resistance value of 8.12 × 10^−2^ Ω/sq was obtained for the 10:10:1 sample. Therefore, a weight ratio of 10:10:1 will be used in subsequent experiments.

### 3.3. Antioxidation and High Conductivity Stability of CuNPs/PTT/GO Composite Films

**Figure 5:**
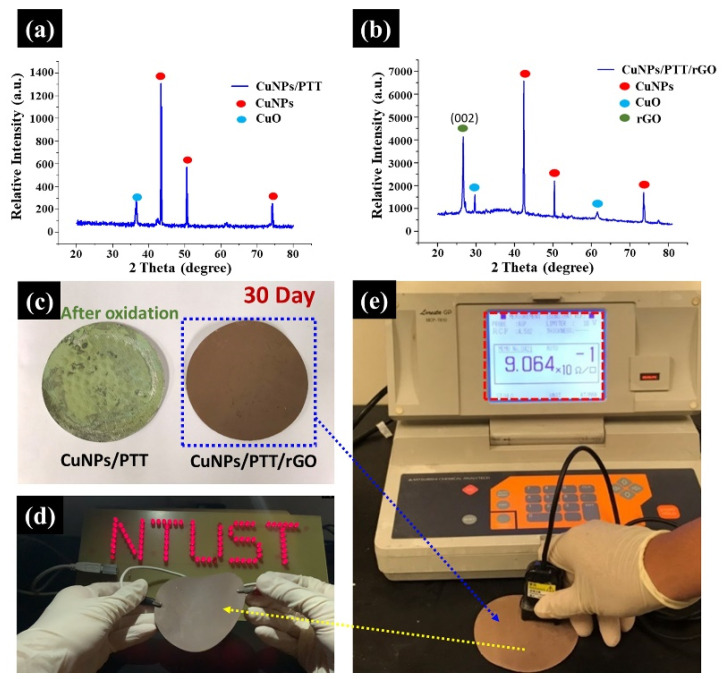
XRD analysis of (**a**) CuNPs/PTT. (**b**) CuNPs/PTT/GO hybrid. (**c**) Digital photos of oxidized CuNPs/PTT and non-oxidized CuNPs/PTT/GO films after 30 days of air exposure. (**d**) The sheet resistance of the CuNPs/PTT/GO film was measured after 30 days of air exposure. (**e**) The LED circuit test of the CuNPs/PTT/GO film demonstrated excellent electrical conductivity.

The sample with the highest conductivity in Table 1 was selected for the anti-oxidation test. A comparison was performed on two groups of CuNPs/PTT/GO films with weight ratios of 1:1:0 and 10:10:1. The films were exposed to the atmosphere for 30 days. Further verification of CuNPs oxidation was obtained through XRD analysis, as shown in Figure 5a. Three diffraction peaks appeared at 43.3°, 50.4°, and 74.2° for CuNPs/PTT corresponding to the (1 1 1), (2 0 0), and (2 2 2) crystal planes of the nano copper, respectively. The peak at 2θ = 36.5° was generated by CuO. According to Figure 5b, when CuNPs/PTT was added with GO, the original CuO peak at 36.5° was significantly reduced and the (0 0 2) crystal plane of GO appeared at 2θ = 26.2°, which confirmed the presence of amorphous carbon in GO. The high-temperature sintering process lowered the anti-oxidation effect of CuNPs. Next, a four-point probe was used to record the changes in resistance. According to the digital images in Figure 5c, after 30 days of exposure to the atmosphere and in the absence of GO coating, the CuNPs/PTT film surface was oxidized; whereas the CuNPs/PTT/GO film remained reddish-brown, indicating that CuNPs/PTT/GO film possesses anti-oxidation properties. A four-point probe was used to measure the surface resistance of the CuNPs/PTT/GO film after 30 days, as shown in Figure 5d. After the CuNPs/PTT, CuNPs/PTT/GO film is placed for 0 days, 7 days, and 30 days, the resistance value of CuNPs/PTT, CuNPs/PTT/GO film is measured through the four-point probe, respectively. The results show that the surface impedance increased from 8.12 × 10^−2^ Ω/sq on the first day to 9.06 × 10^−1^ Ω/sq; and that of CuNPs/PTT increased from 6.92 × 10^−2^ Ω/sq to 3.45 × 10^7^ Ω/sq after 30 days, indicating that the CuNPs/PTT film had lost its electrical conductivity. The LED circuit test is demonstrated in Figure 5e and Video S1 in Supporting Information. The CuNPs/PTT/GO film connected and illuminated multiple LED bulbs in series. As the number of LED bulbs increased, the resistance also increased, and the low resistance of the CuNPs/PTT/GO film allowed the LED bulbs to be maintained at a certain brightness. Video S1 demonstrates the high flexibility of this film.

### 3.4. Signal Measurement with the ECG Smart Clothes

**Figure 6:**
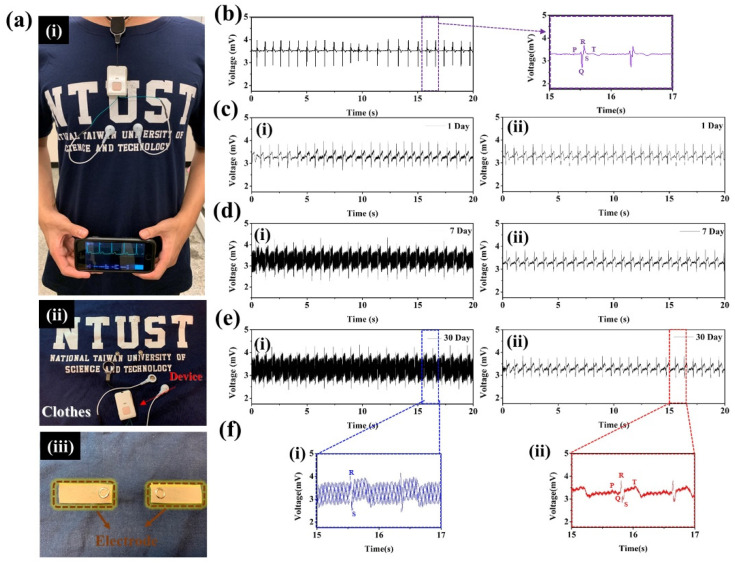
(**a**) (i) Digital image of CuNPs/PTT/GO films on smart clothing. (ii) External structure of ECG clothing, (iii) CuNPs/PTT/GO films on internal image of clothing. ECG signals collected. (**b**) Ag-Ag/Cl electrode and after (**c**) 1 day, (**d**) 7 days, and (**e**) 30 days and (**f**) 30-day signal enlargement, where (i) and (ii) represent CuNPs/PTT and CuNPs/PTT/GO film electrodes, respectively.

**Figure 7:**
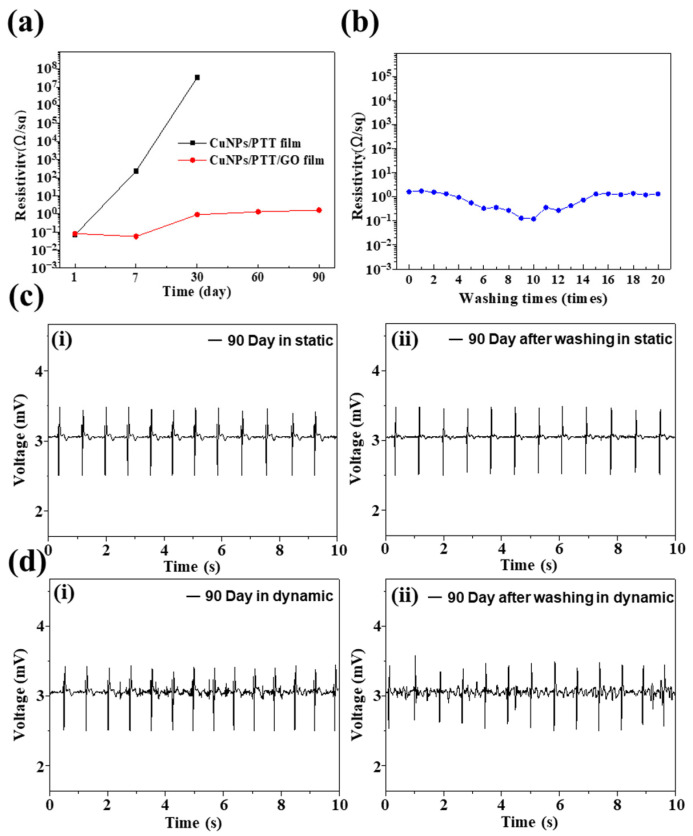
(**a**) The resistance values of CuNPs/PTT film and CuNPs/PTT/GO film for different days, (**b**) The resistance value of CuNPs/PTT/GO film after different times of washing test. (**c**) Static ECG and (**d**) Dynamic ECG signals where (i) before washing test and (ii) after 20 times wash cycles of CuNPs/PTT/GO film electrodes, respectively.

In this section, we describe measurement of the ECG signals using the ECG smart clothes device. The measurement conditions were as follows: with a sampling frequency of 500 Hz, a 20-second resting ECG was recorded from the left chest of a 25-year-old male (Figure 6 and Video S1 in the Supporting Information). The ECG smart clothes device was constructed by processing metal buttons on the CuNPs/PTT film or the CuNPs/PTT/GO film, which were then connected to the ECG device using metal wires. The actual ECG device design and the recorded signals are presented in Figure 6a. Figure 6a (i) signals collected by the ECG smart clothes were transmitted via Bluetooth to the screen of a mobile phone; (ii) data was transmitted to the device via buttons on the outside of the clothes; (iii) for comparison with commercially available Ag-Ag/Cl electrodes, human body signals were collected by the Ag-Ag/Cl and CuNPs/PTT/GO film, and the ECG signals were monitored. It shows the ECG signals recorded on the first day by Figure 6a Ag-Ag/Cl and Figure 6b (i) CuNPs/PTT film and (ii) CuNPs/PTT/GO film. ECG signals obtained from the three films can be clearly observed, as well as variations in the characteristic peaks (P-Q-R-S-T) of the ECG. We then observed the oxidation of Cu, and then performed ECG signal reception for different days. Figure 6c shows that, on the 7th day, the (i) CuNPs/PTT film showed signs of oxidation. Table 2 shows the changes in the surface resistance of CuNPs/PTT and CuNPs/PTT/GO films after 7 days. The results indicate that the surface impedance increased from 6.92 × 10^−2^ Ω/sq to 2.30 × 10^2^ Ω/sq, causing significant noise in the ECG signal. The ECG signals that were recorded after 30 days are presented in Figure 6d. The results show that the surface impedance of the (i) CuNPs/PTT film increased to 3.45 × 10^7^ Ω/sq, and the characteristic peaks of the ECG signal could no longer be discerned. After 30 days of oxidation, the (ii) CuNPs/PTT/GO film exhibited good oxidation resistance, its surface resistance only increased by one order of magnitude, and the ECG signal remained relatively stable. Figure 6e shows the zoomed-in view of the 15–17 s interval on Figure 6d indicating that (i) all ECG characteristic peaks (P-Q-R-S-T) can be observed clearly; (ii) due to the high resistance caused by oxidation, only R-S peaks can be distinguished from the ECG signal, which cannot be used for medical diagnosis. Therefore, the PTT/GO hybrid dispersant can provide effective protection for CuNPs, prevent CuNPs from being oxidized, and maintain its high electrical conductivity, making it suitable for application in ECG smart clothes. After the samples were placed for 60 days and 90 days, the resistance values were sorted in Figure 7a. The results show the resistance value of CuNPs/PTT film cannot be detected by the four-point probe (above 10^8^) after 60 days, while CuNPs/PTT/GO film remains stable at around 10^0^. In practical applications, smart clothes will be used to remove dirt from washing machines after being worn. Therefore, the CuNPs/PTT/GO film was washed after being placed for 90 days to determine the change in resistance before and after washing, as shown in Figure 7b. The result reveals that the resistance value gradually decreases when the number of wash cycles is carried out from 1 to 10 cycles. The resistance value gradually increases and slows down after 10 to 20 cycles. This result is similar to findings of previous literature [49]. This can be attributed to the fact that when the number of washing cycles ranges from 1 to 10, the dirt accumulated during the 90-day period of the CuNPs/PTT/GO film gradually disappears, and the resistance value decreases. After 10 to 20 wash cycles, the part of the graphene on the film’s surface that has a weaker interaction force with the copper mesh gradually peels off, causing the resistance value to rise progressively to 1.3 × 10^0^ and tends to be stable. However, to explore the application value of CuNPs/PTT/GO film, we compared the static and dynamic ECG signals of the CuNPs/PTT/GO film that had been placed for 90 days before and after the washing test, as shown in Figure 7c,d. The results reveal that the ECG signals of the CuNPs/PTT/GO film before and after the washing test are similar under static and dynamic conditions. The dynamic ECG signal is relatively static and messy, but can clearly distinguish P-Q-R-S-T waves. To compare the performance of CuNPs/PTT/GO film, we compared with substrates of the sensing element used in other literature. As shown in Table 2, the results show that CuNPs/PTT/GO film has the conductivity characteristics of the sensing element in terms of the resistance value, and it is re-washable. It can be stored for more than three months, which shows that CuNPs/PTT/GO film has commercial potential.

## 4. Conclusions

In this study, CuNPs/PTT/GO hybrid films were prepared by simple in situ polymerization and used as anti-oxidation conductive ECG electrodes for smart clothes. An analysis of the morphology and structure of CuNPs was performed using TEM and UV-vis spectroscopy. A four-point probe and XRD measurements were used to analyze the conductivity and oxidation resistance of CuNPs/PTT/GO hybrid films. The surface impedance results show that the CuNPs/PTT/GO film with a weight ratio of 10:10:1 had a relatively low surface resistance of 8.12 × 10^−2^ Ω/sq. With the addition of GO, CuNPs exhibits antioxidant properties as the 2D GO nanomaterial is conductive and provides a large surface area for the CuNPs. In addition, the PTT dispersant provides CuNPs with good dispersibility and confers a multilayer structure to CuNPs and GO, thus increasing the charge transport and improving the conductivity and stability of the ECG electrode. CuNPs/PTT/GO hybrid films prepared at 300 °C showed morphological characteristics of surface melting in addition to a phenomenon of surface aggregation that formed a network. The surface resistance of CuNPs/PTT/GO thin film was measured as 8.12 × 10^−2^ Ω/sq. After 30 days, its surface resistance remained at 9.06 × 10^−1^ Ω/sq. In conclusion, this research has developed a CuNPs/PTT/GO hybrid film with excellent characteristics, which can be applied to wearable ECG smart clothes in future applications.

## Figures and Tables

**Table 1:** UV-vis absorption peak intensity, average particle size, and sheet resistance of CuNPs/PTT/GO at different ratios.

CuNPs/PTT/GO	UV-Vis Absorption (nm)	Average CuNPs Sizeby TEM (nm) ^a^	Sheet Resistance (Ω/sq) ^b^
1:1:0	571	63.3	6.92 × 10^−2^
20:20:1	577	35.9	3.45 × 10^0^
10:10:1	579	36.7	8.12 × 10^−2^
5:5:1	585	14.2	9.41 × 10^−2^
1:1:1	598	44.0	4.27 × 10^1^

^a^ Average particle sizes of copper were measured by TEM. ^b^ Solution coating on a glass substrate with 50-μm-thick hybrid film at 300 °C, and the resulting sheet resistance measured using a four-point probe.

**Table 2:** Performance comparison of present hybrid films with those reported in the literature.

Materials	Mathod	Ohmic Resistance (Ohm)	Application	Reference
CuO/carbon fiber fabric	hydrothermal method	0.67	non-enzymatic glucose sensor	[49]
PEDOT:PSS/rGO	pad-dry-cure method	125	electrocardiogram	[50]
PEP/3DPG ^a^	drop-cast	105.3	immunosensing, electrocardiogram recording, and microsupercapacitors (MSCs)	[51]
LIG ^b^	laser-induced	10.6	electrocardiogram	[52]
Carbon fiber/CuNPs	electrochemical synthesized	7.96	electrocardiogram	[53]
AgNPs/PIB-POE-PIB/GO	heated	0.012	electrocardiogram	[6]
CuNPs/PTT/GO	heated	0.0812	electrocardiogram	Current study

^a^ PEP/3DPG was polyaziridine-encapsulated phosphorene (PEP)-incorporated flexible 3D porous graphene (3DPG) ^b^ LIG was laser-induced graphene.

## Data Availability

Not applicable.

## Supplementary Materials

The following are available online at https://www.mdpi.com/article/10.3390/polym14020237/s1, Video S1 showing a demonstration of the LED bulbs illuminated using the highly electrically conductive films and a video depicting a highly sensitive electrocardiogram (ECG) measurement by highly electrically conductive films as flexible electrodes under dynamic activity are provided.
